# MAPLE Processed Nanostructures for Antimicrobial Coatings

**DOI:** 10.3390/ijms232315355

**Published:** 2022-12-05

**Authors:** Ariana Hudiță, Valentina Grumezescu, Oana Gherasim, Alexandru Mihai Grumezescu, Gabriela Dorcioman, Irina Negut, Ovidiu-Cristian Oprea, Bogdan Ștefan Vasile, Bianca Gălățeanu, Carmen Curuțiu, Alina Maria Holban

**Affiliations:** 1 Department of Biochemistry and Molecular Biology, University of Bucharest, 050095 Bucharest, Romania; 2 Lasers Department, National Institute for Lasers, Plasma and Radiation Physics, 409 Atomistilor Street, 077125 Magurele, Romania; 3 Department of Science and Engineering of Oxide Materials and Nanomaterials, Politehnica University of Bucharest, 011061 Bucharest, Romania; 4 Research Institute of the University of Bucharest—ICUB, University of Bucharest, 050657 Bucharest, Romania; 5 Academy of Romanian Scientists, Ilfov No. 3, 050044 Bucharest, Romania; 6 Department of Inorganic Chemistry, Physical Chemistry and Electrochemistry, Faculty of Applied Chemistry and Materials Science, Politehnica University of Bucharest, 1–7 Gheorghe Polizu Street, 011061 Bucharest, Romania; 7 Department of Microbiology and Immunology, Faculty of Biology, University of Bucharest, 91–95 Splaiul Independentei Street, 077206 Bucharest, Romania

**Keywords:** eugenol, magnetite, APTMS, MAPLE, antibiofilm

## Abstract

Despite their great benefits for debilitated patients, indwelling devices are prone to become easily colonized by resident and opportunistic microorganisms, which have the ability to attach to their surfaces and form highly specialized communities called biofilms. These are extremely resistant to host defense mechanisms and antibiotics, leading to treatment failure and device replacement, but also to life-threatening complications. In this study, we aimed to optimize a silica (SiO_2_)-coated magnetite (Fe_3_O_4_)-based nanosystem containing the natural antimicrobial agent, eugenol (E), suitable for MAPLE (matrix-assisted pulsed laser evaporation) deposition as a bioactive coating for biomedical applications. X-ray diffraction, thermogravimetric analysis, Fourier-transform infrared spectroscopy, and transmission electron microscopy investigations were employed to characterize the obtained nanosystems. The *in vitro* tests evidenced the superior biocompatibility of such nanostructured coatings, as revealed by their non-cytotoxic activity and ability to promote cellular proliferation and sustain normal cellular development of dermal fibroblasts. Moreover, the obtained nanocoatings did not induce proinflammatory events in human blood samples. Our studies demonstrated that Fe_3_O_4_ NPs can improve the antimicrobial activity of E, while the use of a SiO_2_ matrix may increase its efficiency over prolonged periods of time. The Fe_3_O_4_@SiO_2_ nanosystems showed excellent biocompatibility, sustaining human dermal fibroblasts’ viability, proliferation, and typical architecture. More, the novel coatings lack proinflammatory potential as revealed by the absence of proinflammatory cytokine expression in response to human blood sample interactions.

## 1. Introduction

Device-related infections are frequently reported in the case of patients undergoing hospitalization or using indwelling devices, due to the opportunistic infection with resistant and biofilm-related pathogens, which are responsible for life-threatening conditions and even death [1,2]. In these patients, the microbial contamination and colonization of the device, infection, and biofilm development, are favored by the direct contact between the device’s material and resident bacteria, patient microbiota, and medical personnel [3,4]. The highest risk for device microbial infection is usually in the first 24–48 h after the implantation surgery [5,6]; therefore, prophylactic antibiotic therapy (which facilitate the selection of resistant pathogens) is utilized in such patients.

A particular case is represented by patients with critical conditions or malnutrition-associated pathologies, whose therapy implies the use of gastrostomy tubes as mandatory alternatives for nutrition and medication. However, due to specific loco-regional implantation, such devices are highly susceptible to opportunistic contamination and colonization phenomena [7,8,9].

In this context, and also by considering the complications of high-dosing systemic antibiotherapy and the continuous increase in drug-resistant pathogens, clinicians and researchers explore alternative efficient antimicrobials, including plant-derived compounds that are able to reduce the selection of resistant bugs, while avoiding harmful effects against both the host and environment [10,11]. Nanostructured materials functionalized with natural compounds proved their potential as efficient antimicrobial agents against most clinically relevant microorganisms, including resistant bacteria (superbugs) and biofilm-embedded microbes [12,13].

Eugenol is the main constituent of clove (*Syzygium aromaticum*) essential oil, and exhibits important antimicrobial [14], antitumor [15,16], anti-oxidant [17,18], and anti-inflammatory effects [19,20]. The antimicrobial activity of eugenol is mainly attributed to its phenolic structure and hydrophobic character. These facilitate the enzymatic inhibition of the cellular membrane and the intracellular generation of reactive species, and also the piercing of the microbial cell envelope, respectively [21,22]. In addition, those compositional and structural features contribute to synergistic antimicrobial effects [23,24] and to anti-biofilm efficiency [25,26]. Although many natural substances have demonstrated significant anti-pathogenic activity, some of their properties, such as volatility, instability, and high doses required for an efficient therapy, currently limit their use in the biomedical practice [27,28].

The novelty of this study consists in designing an eugenol-functionalized magnetite core—porous SiO_2_ shell nanosystem to be used as an efficient antimicrobial and biocompatible coating able to reduce colonization and biofilm formation on medical devices. Since no antibiotics are included, this formulation could help in the fight against resistant pathogens, as there are no reports showing that bacteria could become resistant to natural compounds or nanosystems, especially if they are utilized in very low amounts and only interfere with microbial virulence and ability to communicate and develop biofilms [29].

Our previous studies demonstrated that magnetite nanoparticles significantly potentiate the antimicrobial activity of E [30] and the use of a SiO_2_ matrix may increase the amount of embedded compound, ensure a prolonged effect, and provide controlled release. The core/shell Fe_3_O_4_@SiO_2_ nanosystem is suitable for the design of antimicrobial coatings for medical devices [31,32].

## 2. Results and Discussions

Besides possessing a major affinity for surface functionalization, the nanosized-governed high surface-to-volume ratio of nano-magnetite is also responsible for some unwanted effects (such as agglomeration, chemical reactivity, and oxidation) [33,34], which may impact their biocompatibility and biomedical outcomes. In this respect, the hydroxyl-enriched surface of Fe_3_O_4_ nanoparticles provides the proper grounds for conjugation and grafting of different (bio)molecules and (bio)materials [35,36] to modulate their reactivity and biofunctionality.

In our study, the addition of eugenol within the alkaline precipitation solution resulted in the one-step synthesis and facile and fast surface modification of Fe_3_O_4_ nanoparticles, as the presence of the natural organic agent provided particle stabilization [37,38] and encouraged the formation of uniform particles by controlling their growth, shape, and size [39,40].

**Figure 1:**
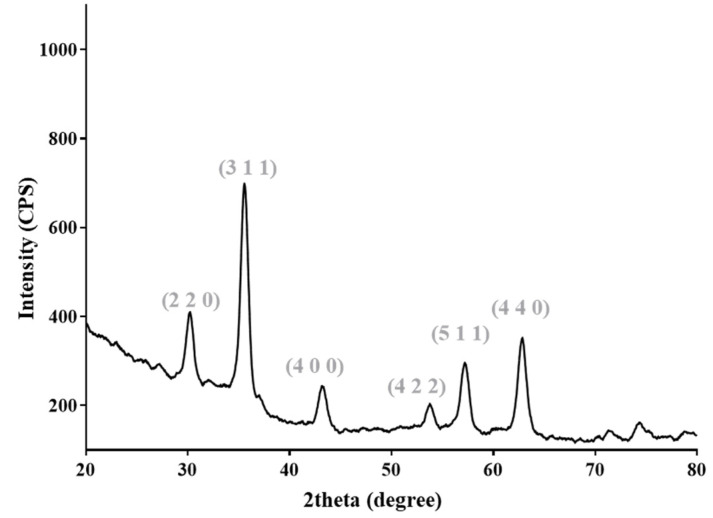
X-ray diffraction pattern of Fe_3_O_4_@E particles.

The XRD pattern included in Figure 1 confirms the formation of cubic structured Fe_3_O_4_ (JCPDS no. 65-3107) [41,42] as the sole mineralogical phase. The strong diffraction interferences evidenced at diffraction angles of ~30, ~35, ~43, ~53, ~57 and ~62 (°) were assigned to crystalline cubic magnetite, corresponding to its specific (2 2 0), (3 1 1), (4 0 0), (4 2 2), (5 1 1), and (4 4 0) diffraction planes [43,44].

**Figure 2:**
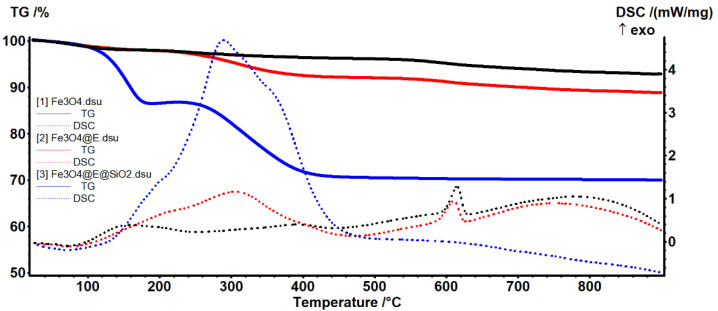
Thermal analysis of Fe_3_O_4_, Fe_3_O_4_@E, and Fe_3_O_4_@E@SiO_2_ particles.

To evaluate the composition of Fe_3_O_4_-based nanoparticles (Fe_3_O_4_@E) and nanosystems (Fe_3_O_4_@E@SiO_2_), thermal studies were performed and correlated with the data corresponding to bare Fe_3_O_4_ sample. Using thermogravimetric analysis (TGA) and differential scanning calorimetry (DSC), both qualitative and quantitative information was obtained (Figure 2).

The pristine sample (Fe_3_O_4_) loses 1.94% of its initial mass up to 160 °C in an endothermic process, with a minimum at 72.5 °C. This can be attributed to the elimination of physically adsorbed water molecules from the sample’s surface. The sample loses 1.94% mass between 160–500 °C in a series of overlapped and weak exothermic processes, with peaks at 193.0 and 393.8 °C. The transformation of magnetite to maghemite takes place in this interval by the oxidation of Fe^2+^ to Fe^3+^ [45], but also the removal of terminal –OH moieties from the nanoparticles surface. After 500 °C, the sample is losing 3.25% of its mass with the exothermic effect from 614.9 °C being specific for the phase transition of maghemite to hematite [46]. The residual mass, brown-reddish hematite, represents 92.87%.

With a similar thermal behavior, the eugenol-functionalized magnetite (Fe_3_O_4_@E)sample loses 1.91% of its initial mass up to 160 °C in an endothermic process with a minimum at 72.4 °C, due to moisture evaporation. Between 160–500 °C, the sample loses 6.08% of its initial mass following a broad and intense exothermic process, with a maximum at 306.7 °C. This event can be attributed to the evaporation and thermal oxidation of the eugenol molecules adsorbed on the nanoparticles. As the complete degradation of eugenol generally occurs below 200 °C [47,48], our results prove the stabilizing role of the magnetite core regarding the volatility of organic phytochemicals. The phase transition of magnetite to maghemite [39,45], but also the removal of terminal hydroxyls [49], also occur in this thermal range. After 500 °C, the sample loses 3.16% of its mass, the residual brown-reddish hematite, representing 88.86%. The exothermic effect from 610.5 °C is specific for the maghemite—hematite transition [46,50].

On the other hand, the silica-coated nanosystem (Fe_3_O_4_@E@SiO_2_) loses 13.32% of initial mass until 215 °C. The process starts with an endothermic effect with a minimum at 72.0 °C that can be attributed to the removal of surface-adsorbed water molecules and decomposition/oxidation of APTMS [51,52]. Moreover, in this interval, at 204.0 °C, the transformation of magnetite to maghemite takes place by oxidation of Fe^2+^ to Fe^3+^ [45,53]. Between 215–500 °C, the sample loses 16.25% of its initial mass. The process is accompanied by a broad, asymmetric, and intense exothermic effect, with a maximum at 289.5 °C, which can be attributed to the oxidation of residuals from APTMS and the formation of the SiO_2_ shell [54,55]. Besides oxidative processes, the removal of terminal –OH moieties from the nanoparticle’s surface also takes place. After 500 °C, the sample loses 0.47% of its mass, the magnetic residue, representing 70.00%. The lack of the sharp exothermic effect within this temperature interval, specific for the phase transition of maghemite to hematite, indicates the presence of the SiO_2_ shell on the nanoparticle’s surface and confirms its protective role and thermal stabilizing effect for nano-magnetite [56,57].

**Figure 3:**
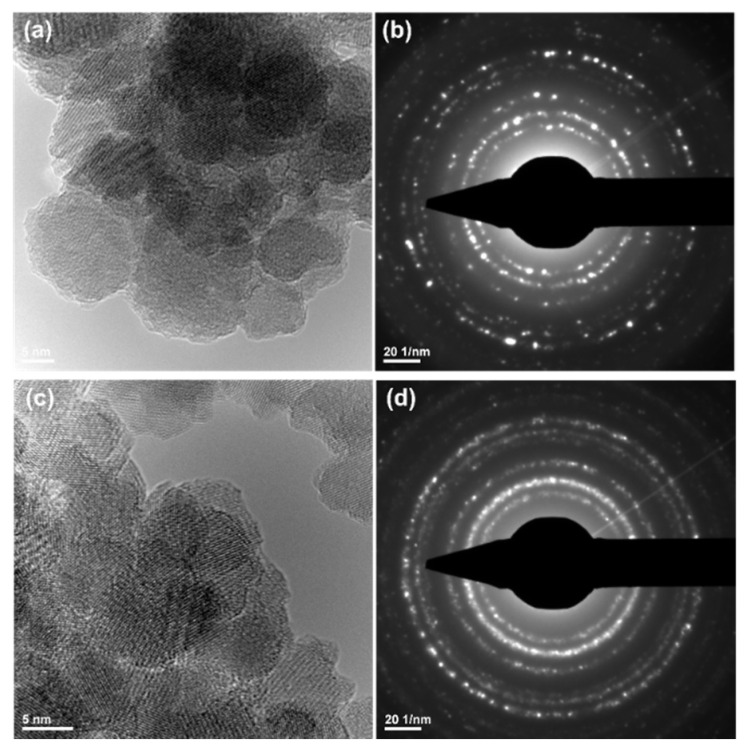
Transmission electron microscopy (**a**,**c**) micrographs and selected area electron diffraction pattern (**b**,**d**) of Fe_3_O_4_@E (**a**,**b**) and Fe_3_O_4_@E@SiO_2_ (**c**,**d**) particles.

The TEM micrographs of synthesized magnetite sample are shown in Figure 3. It can be noticed that Fe_3_O_4_@E and Fe_3_O_4_@E@SiO_2_ particles possess a nanometric size (less than 10 nm) with homogenous particle size distribution and a quasi-spherical shape without aggregation. Structural information on Fe_3_O_4_@E and Fe_3_O_4_@E@SiO_2_ was obtained from SAED analysis (Figure 3b,d). The diffraction ring pattern indicates the polycrystalline nature of the prepared nanostructures, and the identified indices match the lattice facets of cubic magnetite (Fe_3_O_4_) without the presence of any other crystalline phases [58,59].

The Fe_3_O_4_-based nanoparticles and nanosystems were further subjected to MAPLE processing in order to obtain nanostructured thin coatings. In this respect, double-side polished Si substrates and G-tube sections were used as substrates suitable for the physicochemical analysis and *in vitro* evaluation of coatings, respectively.

**Figure 4:**
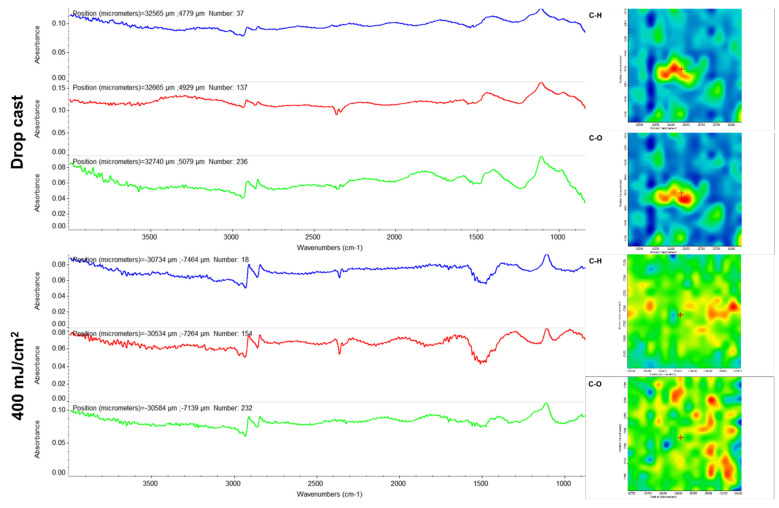
IR spectra (**left**) and IR maps (**right**) of Fe_3_O_4_@E.

In the FT-IR spectra of the initial Fe_3_O_4_ (Figure 4), the bands at ~3400 cm^−1^ and ~1600 cm^−1^ are attributed to phenolic O–H stretching (E) and overlapped benzene C–H stretching (E) and absorbed water molecule vibrations (Fe_3_O_4_), respectively [43,60]. Eugenol shows its signature peaks in the 1100–1250 cm^−1^ region corresponding to C=C and C–O regions [61]. In addition, other peaks corresponding to eugenol (i.e., ~1637, ~1610, and ~1514 cm^−1^, respectively) are due to aromatic C=C stretching [62]. The obtained results are in good agreement with data reported on eugenol [63,64].

Moreover, the FT-IR data corresponding to the Fe_3_O_4_@E coating show the integrity of all previously identified functional groups after MAPLE processing at 400 mJ/cm^2^ laser fluence (Figure 4), with the collected spectra being similar to the dropcast sample. Based on these observations, it can be stated that the MAPLE deposition technique did not damage functional groups or induce changes to the chemical structure of the raw material.

**Figure 5:**
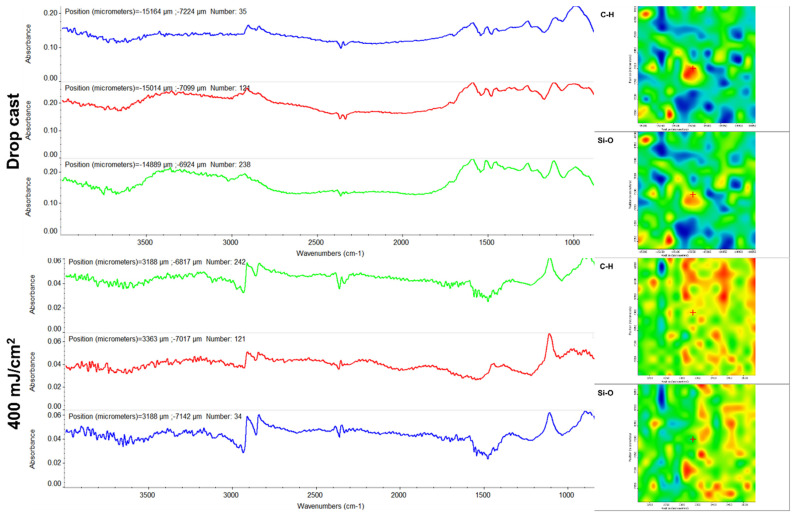
IR spectra (**left**) and IR maps (**right**) of Fe_3_O_4_@E@SiO_2_.

The initial mixture of eugenol and APTMS (Figure 5) presents infrared bands corresponding to the stretching of aromatic C–H (~3072 cm^−1^), =C–H (~2937 cm^−1^), aliphatic C–H (~2836 cm^−1^), aromatic C=C (~2937 cm^−1^), and C–O–C (~1249cm^−1^) originating from E. The infrared maxima identified at ~1051 cm^−1^ and ~912 cm^−1^ result from the overlapped vibrations of E (C–O–C symmetric stretching and benzene C-H out-of-plane bending, respectively) [65] and the silica network (asymmetric Si–O–Si and Si-O-Fe stretching, and symmetric Si–O–Si stretch, respectively) [66,67]. The band from ~1137 cm^−1^ is particularly assigned to the asymmetric stretch of aliphatic amines from APTMS [68].

The infrared spectra of Fe_3_O_4_@E@SiO_2_ coatings (Figure 5, left) evidence the compositional preservation and the unaltered transfer of the initial mixture, while the corresponding infrared maps (Figure 5, right) confirm the laser transfer efficiency of the nanosystems through the highly abundant warm regions, which provide direct information regarding the intensity of the monitored absorbance bands (in our case, C–H and Si–O originating from eugenol and silica, respectively).

**Figure 6:**
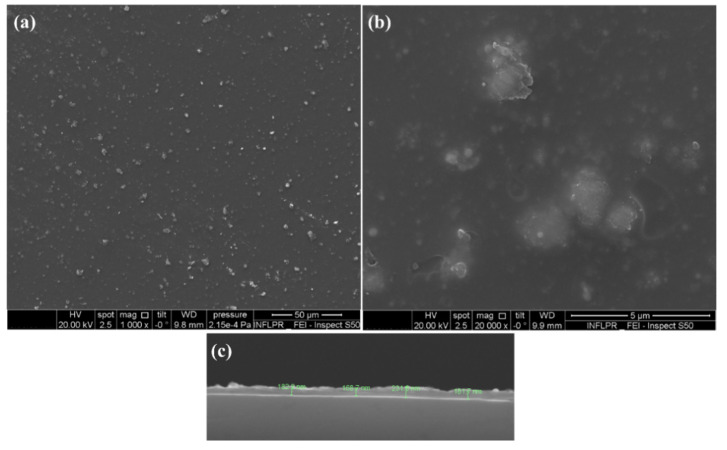
Typical top-view (**a**,**b**) and cross-section (**c**) scanning electron microscopy images of Fe_3_O_4_@E@SiO_2_ coatings.

The surface morphology, structure, and thickness of Fe_3_O_4_@E@SiO_2_ coatings obtained at 400 mJ/cm^2^ laser fluence were evaluated by SEM analysis. The plain-view micrographs (Figure 6a) show the information of a uniform coating which completely cover the substrate and present a particulate aspect and a relatively homogenous aspect. The formation of continuous coatings with irregular surfaces can be further noticed in the cross-section SEM image (Figure 6c), which also reveals that the mean thickness of such nanostructured coatings is ~200 nm.

### 2.1. In Vitro Biocompatibility Assessment

As the coating strategy was employed for tuning G-tubes to improve the current medical devices available for gastroenterology, biocompatibility screening is a mandatory first step in validating materials for their prospective use in the biomedical field. In this view, to assess if the novel coatings display biocompatibility, several parameters of cell health and morphology have been investigated using human dermal fibroblasts as an *in vitro* cellular model.

**Figure 7:**
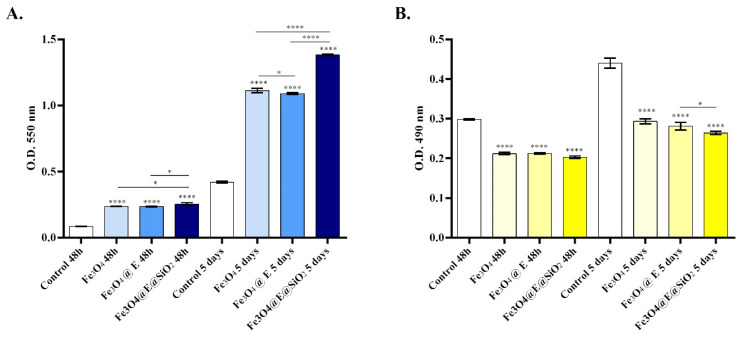
(**A**) MTT assay revealing human dermal fibroblasts CCD-1070Sk viability after 48 h and 5 days post-seeding on the control surface (uncoated sections of catheters) and Fe_3_O_4_, Fe_3_O_4_@E, and Fe_3_O_4_@E@SiO_2_ coatings. (**B**) Sample cytotoxicity profile as revealed by the determined LDH released in the medium samples harvested after 48 h and 5 days from control samples and Fe_3_O_4_, Fe_3_O_4_@E, and Fe_3_O_4_@E@SiO_2_ coatings. Statistical significance: * *p* < 0.05, **** *p* < 0.0001.

The MTT assay showed an overall positive impact of the coatings on human dermal fibroblast cell viability during 5 days of culture (Figure 7A). After 48 h of culture, all coated samples presented a statistically significant (**** *p* < 0.0001) increase in cell viability compared with the non-coated control. At this time point, the highest rate of cell viability was noticed for the Fe_3_O_4_@E@SiO_2_ coatings, followed by the Fe_3_O_4_@E coatings, showing that the addition of eugenol or APTMS in the structure of the coatings does not impact negatively on cellular metabolic health. More, a similar profile of cell viability resulted after 5 days of human dermal fibroblast contact with the novel coatings where all the investigated samples exhibited a statistically significant (**** *p* < 0.0001) increase in cell viability in comparison with the control sample. While CCD-1070Sk cell viability significantly increased post-culture on simple or eugenol-loaded Fe_3_O_4_ coatings, the highest cell viability profile was observed for cells cultured on Fe_3_O_4_@E@SiO_2_ coatings, revealing that this coating sustains the best cell viability. More, despite the capacity of human dermal fibroblasts to proliferate on top of all the tested samples as revealed by the statistically significant increase in cell viability between 5 days and 48 h, the highest proliferation rate was on the Fe_3_O_4_@E@SiO_2_ coatings, followed by a moderate proliferation rate on Fe_3_O_4_ and Fe_3_O_4_ @E coatings. The LDH assay results (Figure 7B) were in full accordance with the MTT assay results as low levels of LDH were released in the culture medium by human dermal fibroblasts seeded on coated samples compared with the uncoated control. After 48 h of cell-coatings contact, all samples presented similar levels of LDH leakage, levels that were statistically significantly lower (**** *p* < 0.0001) compared with the LDH leakage registered in the control sample. Moreover, after 5 days, a similar cytotoxicity profile was found, with the most prominent decrease in the amount of LDH released into the culture being registered in the media samples retrieved from the Fe_3_O_4_@E@SiO_2_ coatings.

**Figure 8:**
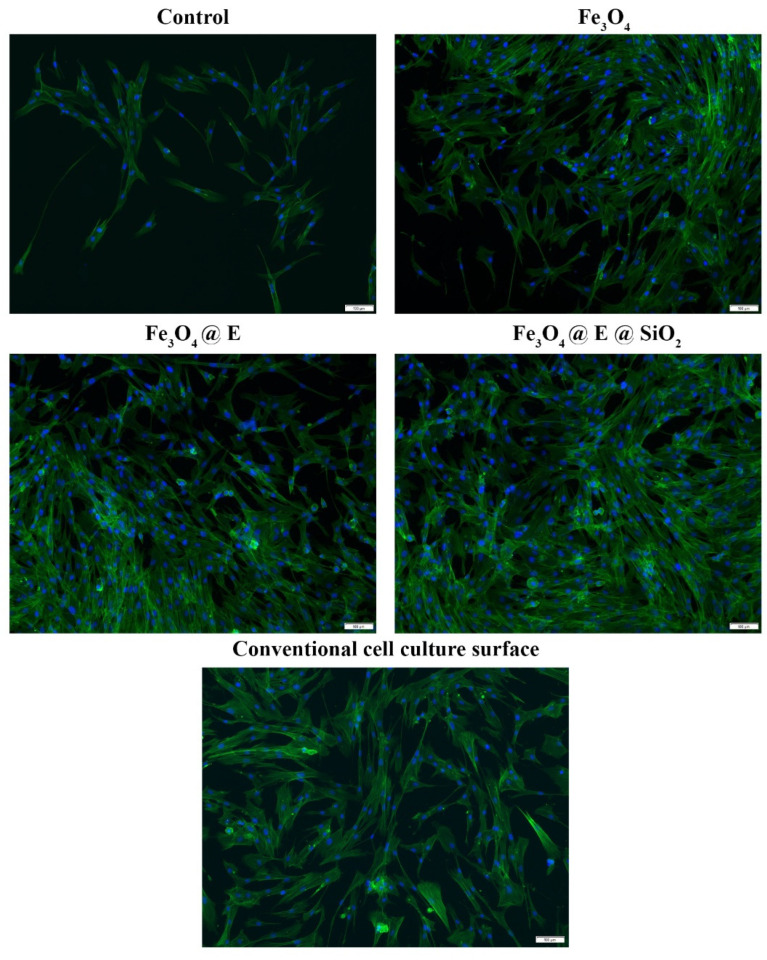
Fluorescence micrographs showing human dermal fibroblast cytoskeletons after 48 h of contact with uncoated samples and Fe_3_O_4_, Fe_3_O_4_@E, and Fe_3_O_4_@E@SiO_2_ coatings. Conventional culture surface (plastic) was used as the control. The actin filaments were stained with phalloidin-FITC (green) and cell nuclei with DAPI (blue). Scale bare: 100 µm.

The possible morphological alterations induced in human dermal fibroblast cytoskeleton 48 h post-contact with the coatings as well as cell distribution on the materials were investigated using fluorescence microscopy (Figure 8). The obtained results showed that no morphological alterations are triggered in response to coating contact. In all analyzed samples, the cells, therefore, presented a well-organized cytoskeleton, followed by human dermal fibroblasts’ spindle-like morphology. This was similar to the morphology observed post-contact with the conventional cell culture surface. More, all cells presented cytoplasmic extensions and filopodia, aspects that suggest that cells are capable to adhere to all the analyzed samples, with more prominent extensions observed in cells cultured on coated samples. On the Fe_3_O_4_@E and Fe_3_O_4_@E@SiO_2_ coatings, cells presented more elongated actin filaments and generated 3D cellular networks despite the fact that no notable differences were observed regarding cell architecture. Moreover, a higher ratio of cells was noticed on coated samples compared with the control. Thus, the cells were distributed evenly on the coating surface, especially on Fe_3_O_4_@E and Fe_3_O_4_@E@SiO_2_ coatings. These results could suggest that human fibroblasts adhere preferentially to coated-samples compared with pristine glass.

All the *in vitro* biological investigations revealed a proper biocompatibility profile for all coated samples as all analyses revealed a non-cytotoxic profile. Moreover, the coatings supported cell viability and proliferation and sustained cellular normal morphology and development. The best cell viability and proliferation potential were evidenced for the Fe_3_O_4_@E@SiO_2_ coatings. These results were clearly depicted after 5 days of culture. While Fe_3_O_4_ and SiO_2_ are intensively used for biomedical applications due to their excellent biocompatibility [69,70], the use of eugenol remains debatable due to its high cytotoxicity exhibited in cell culture models such as human osteoblast, fibroblasts, or endothelial cells even at low concentrations [71,72,73]. However, eugenol is a versatile compound with numerous beneficial effects such as antioxidant, antimicrobial, anticancer, and anti-inflammatory activity [74]. Our results show that, by embedding eugenol in Fe_3_O_4_@E and Fe_3_O_4_@E@SiO_2_ coatings, its cytotoxicity can be overcome since no harmful effects were observed on human dermal fibroblasts seeded on eugenol-based samples.

### 2.2. Ex Vivo Proinflammatory Potential Assessment

**Figure 9:**
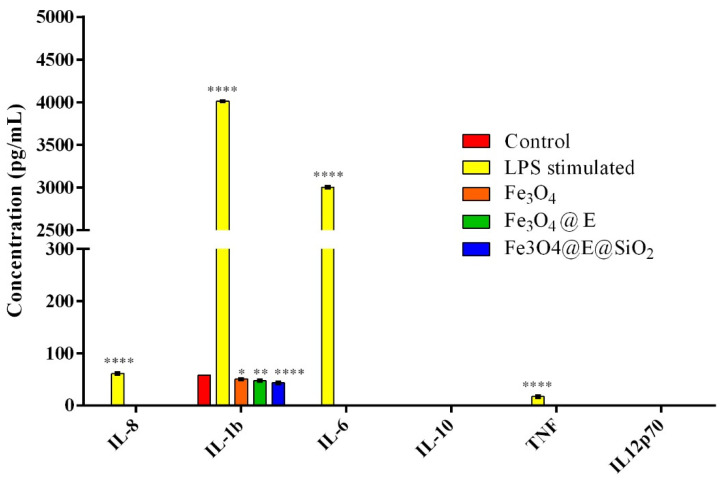
Graphical representation of interleukin-8 (IL-8), interleukin-1β (IL-1β), interleukin-6 (IL-6), interleukin10 (IL-10), tumor necrosis factor (TNF), and interleukin12p70 (IL-12p70) levels detected after 6 h and 24 h by flow cytometry in serum isolated from whole blood samples stimulated with LPS, uncoated samples (control), and Fe_3_O_4_, Fe_3_O_4_@E, and Fe_3_O_4_@E@SiO_2_ coatings. Statistical significance: * *p* < 0.05, ** *p* < 0.01, **** *p* < 0.0001.

Very often, medical devices trigger the initiation of the inflammatory response, therefore investigation of novel materials’ proinflammatory potential could better characterize an original material designed for biomedical applications and validate its feasibility to being implemented. Whole blood samples can be used for investigating cytokine production in response to a proinflammatory stimulus [75]. In this view, the expression of six relevant proinflammatory cytokines was investigated in blood samples harvested from healthy donors exposed to the original coatings by flow cytometry. After 6 h of blood-samples interaction, no positive expression of any of the six analyzed cytokines was identified in any of the experimental samples. More, no positive expression was identified in response to LPS blood stimulation; the production of all six cytokines was not identified, in this case, only interleukin-1β (IL-1β) and tumor necrosis factor (TNF) were expressed at low levels of 10.16 pg/mL and 3.40 pg/mL respectively. After 24 h of whole blood stimulation with LPS or the experimental samples (Figure 9), a statistically significant increase in the cytokine levels of IL-1β and TNF was observed compared with 6 h in samples stimulated with LPS. In LPS-stimulated whole blood for 24 h, IL-1β was induced to 4013.76 pg/mL and TNF to 17 pg/mL, while cytokines that were not detectable at 6 h showed elevated levels at this time point: 3004.97 pg/mL for IL-6 and 61.16 pg/mL for IL-8. In comparison, the control sample and coatings triggered the sole expression of IL-1β from all the analyzed cytokines, detected at statistically significantly lower concentrations in all samples compared with the LPS-stimulated samples. The highest levels of IL-1β were detected in whole blood exposed to non-coated samples (58.32 pg/mL), followed by Fe_3_O_4_ coatings (50.57 pg/mL), Fe_3_O_4_@E coatings (47.65 pg/mL), and Fe_3_O_4_@E@SiO_2_ coatings (43.55 pg/mL). This pattern could be attributed to the anti-inflammatory potential of eugenol [76] since the lowest concentrations were detected in eugenol-loaded samples. However, taking together the low concentrations of IL-1β identified in response to non-coated and coated samples’ blood exposure, with the lack of expression of the other proinflammatory cytokines, the results indicate that the coatings do not exhibit proinflammatory effects.

### 2.3. Antibacterial Efficiency

The growth of planktonic (free-floating) microorganisms is important in the evaluation and management of infections. It has been recently suggested that high-density bacterial growth is critical for the progression of infection towards a chronic state. This aspect seems to be more important than bacterial virulence and even biofilm formation for the fitness of infecting bacteria in some infections, such as chronic wounds [77]. Our results showed that the tested coatings present slightly different antimicrobial effects depending on the type of bioactive agent and time of exposure. Thus, after 24 h incubation in nutritive media, no significant growth changes were observed in the analyzed samples. However, a slight planktonic growth impairment is observed for the Eugenol (E)-containing samples after 48 h and 72 h. This could be related to the proved antimicrobial activity of this plant-derived compound [78].

**Figure 10:**
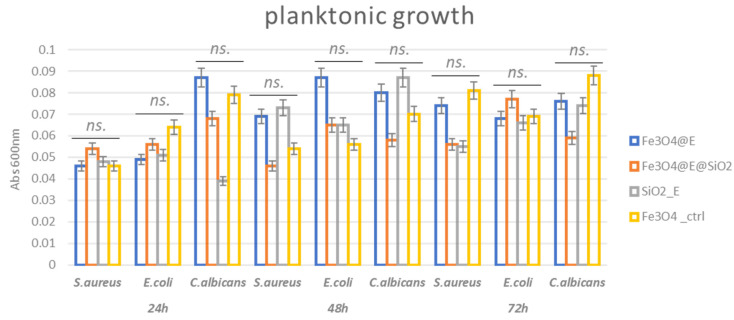
Graphic representation of planktonic growth results. Statistical significance: *ns.* = not significant.

The fact that planktonic cell growth is not significantly inhibited (Figure 10) could be related to a low release rate of the bioactive compound (E) in the culturing media. The released amount could be too low to reach inhibitory concentrations in the culture media in the tested time frame. However, the presence of the bioactive compound in the coating inhibits the attachment of microbial cells on the coating.

**Figure 11:**
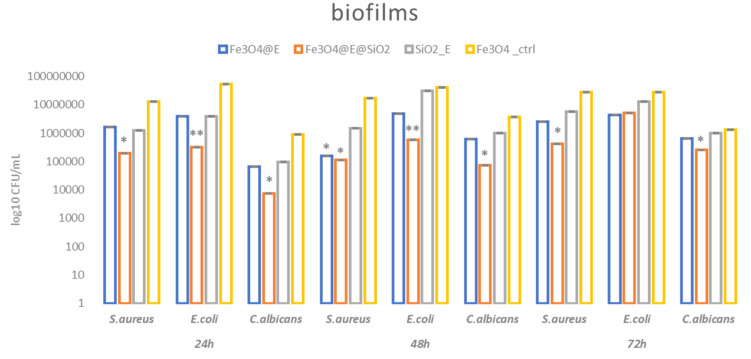
Graphic representation of microbial biofilms developed for 24 h, 48 h, and 72 h on the designed coatings. Statistical significance: * *p* < 0.05, ** *p* < 0.01, when comparing bioactive coatings against control (coating containing plain Fe_3_O_4_ nanoparticles).

Even though the planktonic growth is not significantly inhibited, we observed better inhibition in biofilm development. As expected, monospecific microbial biofilms were mostly inhibited by the Fe_3_O_4_@E@SiO_2_ coatings, and this effect is observed in all the evaluated strains and tested time periods (with the exception of *E. coli* at 72 h). However, the highest inhibition potential of bacterial attachment and biofilm formation on the coating is observed in the first two days of evaluation. Moreover, significant biofilm inhibition was seen in the presence of the other types of coatings containing eugenol (namely: Fe_3_O_4_@E), especially after 24 h and 48 h of incubation (Figure 11).

The better antimicrobial effect of Fe_3_O_4_@E@SiO_2_ coating could be explained by the fact that silica (SiO_2_) could offer a matrix that would ensure controlled and prolonged release of the bioactive agent [79] (E) linked with the magnetite nanoparticles [80]. Moreover, the presence of the nanoparticles could elicit a more significant antibacterial effect of E, as it was previously reported that Fe_3_O_4_ nanoparticles could improve the activity of essential oils and volatile plant extracts [81,82].

## 3. Materials and Methods

### 3.1. Materials

All reagents used to obtain the nanosystems and nanostructured coatings were purchased from Sigma-Aldrich (Merck Group, Darmstadt, Germany), if not specified, namely, anhydrous ferric chloride (FeCl_3_, >99.99%), ferrous sulfate heptahydrate (FeSO_4_·7H_2_O, >98%), dimethyl sulfoxide (DMSO), 3-amino propyl trimethoxysilane (APTMS, 97%), and eugenol (99%). For the biological investigations, most reagents were purchased from Sigma/Merck (Steinheim, Germany), except for the fetal bovine serum (Gibco, ThermoFischer Scientific, Waltham, MA, USA) and the BD CBA Human Inflammatory Cytokines kit (Franklin Lakes, Becton Dickinson, NJ, USA).

### 3.2. Synthesis Methods

#### 3.2.1. Synthesis of Fe_3_O_4_-Based Nanosystems

Magnetite nanoparticles functionalized with Eugenol (Fe_3_O_4_@E) were obtained using a chemical co-precipitation method, in compliance with previous studies [83,84], which reported that spherical-shaped and nanosized Fe_3_O_4_ particles can be thus obtained. The metallic precursor solution obtained by FeCl_3_ and FeSO_4_·7H_2_O in deionized water was added drop by drop within an alkaline solution containing 25% ammonium hydroxide (NH_3_·OH) and E. The process was completed under magnetic stirring and the resultant precipitate was washed several times with deionized water under magnetic separation. The final product was dried at 40 °C for 6 h under an inert atmosphere. To prepare the silica shell, we used the same protocol but added APTMS within the alkaline solution and the resultant core/shell nanosystems were further noted as Fe_3_O_4_@E@SiO_2_

#### 3.2.2. Synthesis of Fe_3_O_4_-Based Coatings

The MAPLE targets were prepared by freezing the suspensions of Fe_3_O_4_@E and core/shell nanosystems (2%) in DMSO at liquid nitrogen temperature.

The as-obtained solid targets were further irradiated with a KrF* excimer laser (λ = 248 nm, τ_FWHM_ = 25 ns) using a COMPexPro 205 Lambda Physics source from Coherent (Göttingen, Germany). Experimental MAPLE parameters were set as follows: room temperature and 0.1 Pa pressure inside the deposition chamber, 4 cm target-to-substrate distance, 0.4 Hz and 15 Hz target rotation and laser repetition frequency, respectively. A total number of 100,000 laser pulses was applied at 400 mJ/cm^2^ laser fluence to obtain Fe_3_O_4_@E and Fe_3_O_4_@E@SiO_2_coatings. All coatings were obtained on sections of catheter (1 cm length) and polished Si substrates (1 cm^2^ area).

### 3.3. Physicochemical Investigation

#### 3.3.1. X-ray Diffraction (XRD)

The purity and crystallinity of the synthesized powdery sample were evaluated by grazing incidence XRD analysis, which was performed using the Cu_Kα_ radiation (λ = 1.541874 Å) of a PANalytical Empyrean diffractometer (Almelo, The Netherlands) equipped with a 2 × GE (2 2 0) hybrid monochromator for Cu and a parallel plate collimator on the PIXcel3D detector(Malvern Panalytical, Malvern, UK). The scans were collected in the 20–80° diffraction angle range, with 0.5° incidence angle, 0.04° step dimension, and 3 s time step.

#### 3.3.2. Thermogravimetric Analysis (TGA)

The thermal behavior of pristine, E-functionalized nanoparticles and silica-coated nanosystems was analyzed using a Shimadzu DTG-TA-50H equipment (Carlsbad, CA, USA). The tests were performed in normal atmosphere by heating small amounts of all powdery samples from room temperature up to 1000 °C, with a heating rate of 1 °C/min.

#### 3.3.3. Transmission Electron Microscopy (TEM)

The TEM data were collected using the Tecnai^TM^ G2 F30 S-TWIN high-resolution transmission electron microscope equipped with selected area electron diffraction (SAED) accessory from FEI Company (Hillsboro, OR, USA). During data collection, which was performed in the transmission mode, the specific point and line resolutions of the microscope were 2 Å and 1 Å, respectively. Before TEM investigation, small amounts of the Fe_3_O_4_ sample were dispersed in ethanol, sonicated for 15 min, and then placed onto the carbon-coated copper grid and dried at room temperature.

#### 3.3.4. Infrared Microscopy (IRM)

IRM analysis, which included the collection of complementary Fourier-transform infrared spectroscopy (FT-IR) spectra and infrared maps, was performed to investigate the stoichiometry and composition, as well as to evaluate the laser transfer efficiency of all nanostructured coatings obtained by MAPLE, respectively. A Nicolet iN10 MX FT-IR microscope (Thermo Fischer Scientific Company, Waltham, MA, USA), operated in the transmission mode in the 5000–500 cm^−1^ wavenumber range, was used in this respect. The scans (40 individual measurements/each sample) were collected at 4 cm^−1^ resolution, and they were further processed using the OmincPicta 8.0 software (Thermo Fischer Scientific Company).

#### 3.3.5. Scanning Electron Microscopy (SEM)

The morphological features of the experimental MAPLE coatings were evaluated by SEM analysis, which was performed using an Inspect S scanning electron microscope (FEI Company, Eindhoven, The Netherlands). The micrographs were collected at 20 kV acceleration voltage of the secondary electron beam, after all samples were capped with a thin gold layer (to diminish the accumulation of electric charges).

### 3.4. Biological Investigations

#### 3.4.1. *In Vitro* Biocompatibility Screening

To investigate the biocompatibility of the novel coatings, the human dermal fibroblast CCD-1070Sk (ATCC^®^ CRL-2091™) cell line was used. Briefly, CCD-1070Sk cells were cultured all throughout the experiments in Dulbecco’s Modified Eagle’s Medium (DMEM, Sigma/Merck, Steinheim, Germany) supplemented with 10% fetal bovine serum (FBS, Gibco, Thermo Fischer Scientific, Waltham, MA, USA) and 1% penicillin-streptomycin mixture (Sigma/Merck) and maintained under standard cell culture conditions (5% CO_2_, 37 °C) in a humidified atmosphere. Prior to experiment initiation, coatings were sterilized by UV exposure for 20 min and then transferred under aseptic conditions in 12-well cell culture plates. After sterilization, human dermal fibroblasts were seeded on top of the coatings at an initial density of 2 × 10^4^ cells/cm^2^ and incubated for 2 h to allow cellular attachment. Uncoated sections of catheters were used as experimental controls for all studies and processed identically as coated samples for all *in vitro* assays. Afterward, samples were immersed in a complete culture medium and incubated for 5 days in cell culture standard conditions. After 48 h and 5 days of culture, samples were processed for the following tests to reveal their biocompatible profile: MTT assay, LDH assay, Live/Dead assay, and staining of actin filaments.

The MTT (3-[4,5-dimethylthiazol-2-yl]-2,5 diphenyl tetrazolium bromide) reduction assay was employed to assess the cellular metabolic activity of CCD-1070Sk cells to highlight the capacity of the original coatings to sustain cellular viability and proliferation. For this, the cell culture medium was discarded and replaced with a freshly prepared solution of 1 mg/mL MTT (Sigma/Merck). Following 4 h incubation in the dark at 37 °C, the obtained formazan crystals were subsequently dissolved in isopropanol (Sigma/Merck) and the optical densities (OD) of the resulting solutions were measured at 550 nm using the multimodal reader FlexStation III (Molecular Devices, San Jose, CA, USA).

The cytotoxic potential of the novel coatings was evaluated using the LDH release assay. In this view, medium samples harvested at the two experimental time points were mixed with the components of the “*In vitro* toxicology assay kit lactate dehydrogenase based TOX—7” kit (Sigma/Merck) according to the manufacturer’s recommendations. After 30 min of incubation at RT in the dark, the absorbance of the resulting solutions was read at 490 nm using the multimodal reader FlexStation III, with the OD being directly correlated with cell membrane damage and cell death.

Finally, to evaluate the overall behavior of human dermal fibroblasts and the possible cytoskeleton alterations induced by coating contact, the F-actin filaments were stained with phalloidin. Briefly, samples were retrieved from the culture plates, washed with PBS (Sigma/Merck), and fixed for 15 min using a 4% paraformaldehyde solution (Sigma/Merck). Then, the cell membrane was permeabilized using a 0.1% Triton X-100 solution in 2% BSA (Sigma/Merck) and 30 min incubation at RT. For revealing the cytoskeleton actin filaments, samples were incubated in the dark for 1 h at 37 °C with a phalloidin-FITC solution (Sigma/Merck). Prior to microscopy investigation, cell nuclei were stained with 4′,6-diamidino-2-phenylindole (DAPI) and samples were analyzed using the Olympus IX73 fluorescence microscope (Olympus Corporation, Tokyo, Japan) and CellSense F software version 1.11.

All the experiments were performed in triplicate and all the statistical data are presented as mean values ± standard deviation of three independent experiments. For statistical analyses, GraphPad Prism software (San Diego, CA, USA) was employed using either one or two-way analyses of variance (ANOVA), with Bonferroni’s multiple comparisons post test used to identify which groups were different. Results with *p* < 0.05 were considered statistically significant.

#### 3.4.2. Proinflammatory Potential Assessment

The proinflammatory potential of the original coatings was evaluated using blood samples harvested from healthy donors that were incubated for 24 h with the samples. The experimental control was represented by uncoated sections of the catheter. For the positive control, blood was stimulated with lipopolysaccharide from *Escherichia coli* O111:B4 (LPS, 10 μg/mL, Sigma/Merck). After 6 h and 24 h of blood-coating interactions, the blood samples were centrifuged and plasma supernatant was further processed to quantify the expression of multiple cytokines using a bead-based multiplex assay (BD CBA Human Inflammatory Cytokines kit, Becton Dickinson, Franklin Lakes, NJ, USA). The following cytokine protein levels were investigated: interleukin-8 (IL-8), interleukin-1β (IL-1β), interleukin-6 (IL-6), interleukin10 (IL-10), tumor necrosis factor (TNF), and interleukin12p70 (IL-12p70). For this, 50 µL of the plasma samples were incubated for 2 h at RT and darkness with 50 µL of IL-8, IL-1β, IL-6, IL-10, TNF, and IL-12p70 mixed Capture Beads and with 50 µL Inflammation PE Detection Reagent. After a wash step, all tubes were analyzed in a Gallios flow cytometer (Beckman Coulter, Brea, CA, USA) using Kaluza for data processing and analysis. All the samples for creating the standard curve were processed identically. The graphical representation of the obtained results was performed using GraphPad Prism software. The mean of the obtained data was obtained from three independent experiments and is presented as the arithmetic mean ± S.D. The statistical significance (* *p* ≤ 0.05) was determined using the two-way ANOVA algorithm for group comparison, Bonferroni test.

#### 3.4.3. Antimicrobial Assessment—Inhibition of Planktonic Growth

To evaluate the antimicrobial efficiency of the coatings, we utilized three microbial strains recognized as models for device infections caused by Gram-positive (*Staphylococcus aureus* ATCC^®^ 25923), Gram-negative (*Escherichia coli* ATCC^®^ 25922), and yeast (*Candida albicans* ATCC^®^ 10231) strains. For the planktonic growth analysis, UV sterilized uncoated (control) and coated catheter sections were aseptically placed in a well of a sterile 24-well plate. Over the specimens, 1 mL of liquid medium (nutritive broth for bacteria and YPG (yeast peptone glucose) for yeast) was added. Then, 10 μL of 0.5 McFarland density (1.5 × 10^8^ CFU (colony forming units)/mL) microbial suspensions prepared in PBS (phosphate buffered saline) was added. The prepared plates were incubated at 37 °C for 24 h. After the incubation, the optical density (OD) of the microbial cultures (absorbance, Abs 600 nm) was measured spectrophotometrically [85].

#### 3.4.4. Antimicrobial Assessment—Modulation of Biofilm Formation

In this assay we evaluated short-term and long-term antimicrobial efficiency against monospecific biofilms. The antibiofilm efficiency was established by evaluating the bacterial biofilm development in the presence of the obtained coatings. The UV sterilized specimens were placed in sterile 24-well plates in 1 mL nutritive liquid media, followed by the inoculation of 10 μL of microbial suspension of 0.5 McFarland standard density from each microbial strain. The as-prepared plates were incubated for 24 h at 37 °C. After incubation, culture media was removed and the samples were washed with 1 mL sterile PBS to remove the unattached bacteria. The catheter samples containing attached bacteria were then transferred to sterile 24-well plates containing fresh media and incubated for 24 h, 48 h, and 72 h, respectively, at 37 °C to allow the growth of monospecific biofilms resulting from the multiplication of attached cells. After incubation, the catheter samples were gently washed with sterile phosphate-buffered saline and further placed in 1.5 mL centrifuge tubes containing 1000 μL of PBS. The obtained specimens were vortexed for 30 s and subsequently subjected to ultrasounds for 10 s to detach the biofilm cells and obtain microbial suspensions of cells which were previously embedded into biofilms. Serial ten-fold dilutions were performed from the obtained suspensions and inoculated on nutrient agar for viable cell counts assay. All the experiments were performed in triplicate and repeated on three separate occasions [85]. Antimicrobial results were analyzed using the one-way ANOVA repeated measures test. All statistical analyses were performed using GraphPad Prism Software, v. 5.03 (GraphPad Software, La Jolla, CA, USA).

## 4. Conclusions

In this study, we optimized a magnetite and silica-based coating containing a natural bioactive compound with antimicrobial properties, namely, eugenol, suitable for MAPLE deposition. The core/shell Fe_3_O_4_@SiO_2_ nanosystems were designed to address the propensity of the indwelling devices to become colonized by microorganisms, thus triggering medical device-associated infections. Moreover, these infections are extremely resistant to host defense mechanisms and antibiotics, therefore, hindering widespread use of medical devices in medical practice. Our results showed that the Fe_3_O_4_@SiO_2_@E presents excellent biocompatibility, sustaining human dermal fibroblasts viability, proliferation, and typical cell architecture. In contact with human blood, the core/shell Fe_3_O_4_@SiO_2_ nanosystems did not stimulate the secretion of the investigated proinflammatory cytokines, revealing that the coating does not exhibit proinflammatory effects. The efficient anti-biofilm properties of the coating were manifested in Gram-positive and Gram-negative opportunistic bacteria, but also, yeast. Taken together, the obtained results sustain the potential of the Fe_3_O_4_@SiO_2_ nanosystems loaded with the natural compound eugenol to be further explored as a coating strategy for indwelling devices to decrease the medical device-associated infection statistics. However, further *in vivo* studies need to be performed to validate the novel coatings.

## Data Availability

Not applicable.
